# Investigating the utility of VR for spatial understanding in surgical planning: evaluation of head-mounted to desktop display

**DOI:** 10.1038/s41598-021-92536-x

**Published:** 2021-06-29

**Authors:** Georges Hattab, Adamantini Hatzipanayioti, Anna Klimova, Micha Pfeiffer, Peter Klausing, Michael Breucha, Felix von Bechtolsheim, Jens R. Helmert, Jürgen Weitz, Sebastian Pannasch, Stefanie Speidel

**Affiliations:** 1 Division of Translational Surgical Oncology (TSO), National Center for Tumor Diseases (NCT), Partner Site Dresden, 01307 Dresden, Germany; 2 Centre for Tactile Internet with Human-in-the-Loop (CeTI), TU Dresden, 01062 Dresden, Germany; 3 Unit of Lifespan Developmental Neuroscience, Faculty of Psychology, TU Dresden, 01062 Dresden, Germany; 4 Institute for Medical Informatics and Biometry (IMB), Faculty of Medicine, TU Dresden, 01307 Dresden, Germany; 5 Department of Visceral, Thoracic and Vascular Surgery, Faculty of Medicine, University Hospital Carl Gustav Carus, Dresden, Germany; 6 Engineering Psychology and Applied Cognitive Research, Faculty of Psychology, TU Dresden, 01062 Dresden, Germany; 7 Core Unit for Data Management and Analytics, National Center for Tumor Diseases (NCT), Partner Site Dresden, 01307 Dresden, Germany

**Keywords:** Biomedical engineering, Surgical oncology, Information technology

## Abstract

Recent technological advances have made Virtual Reality (VR) attractive in both research and real world applications such as training, rehabilitation, and gaming. Although these other fields benefited from VR technology, it remains unclear whether VR contributes to better spatial understanding and training in the context of surgical planning. In this study, we evaluated the use of VR by comparing the recall of spatial information in two learning conditions: a head-mounted display (HMD) and a desktop screen (DT). Specifically, we explored (a) a scene understanding and then (b) a direction estimation task using two 3D models (i.e., a liver and a pyramid). In the scene understanding task, participants had to navigate the rendered the 3D models by means of rotation, zoom and transparency in order to substantially identify the spatial relationships among its internal objects. In the subsequent direction estimation task, participants had to point at a previously identified target object, i.e., internal sphere, on a materialized 3D-printed version of the model using a tracked pointing tool. Results showed that the learning condition (HMD or DT) did not influence participants’ memory and confidence ratings of the models. In contrast, the model type, that is, whether the model to be recalled was a liver or a pyramid significantly affected participants’ memory about the internal structure of the model. Furthermore, localizing the internal position of the target sphere was also unaffected by participants’ previous experience of the model via HMD or DT. Overall, results provide novel insights on the use of VR in a surgical planning scenario and have paramount implications in medical learning by shedding light on the mental model we make to recall spatial structures.

## Introduction

In every day life, finding objects requires spatial navigation and memorizing their relative position with respect to external cues^1^. This mechanism is also used in the context of surgical planning and structural anatomy, where surgeons and medical students are required to learn the internal structure of the organs, as well as their spatial relations to other organs. Classical approaches for the teaching of the human gross anatomy relies on books with detailed illustrations of anatomical structures, life-size plastic organ models, and practical dissection courses on body donors. In order to be able to distinguish which organs contribute to which systems and where they are located, medical students study different anatomy systems (e.g., circulatory, nervous, lymphatic, etc) as well as the intrinsic organ features of these systems, such as the number of segments in a liver^2^. In the particular case of surgical oncology, the focus shifts to the management of cancerous tumors, especially malignant tumors. Before surgery, image data such as a computed tomography (CT) scan is acquired, which includes the location of the tumor and surrounding tissue. As part of surgical planning, surgeons rely on this data and typically create a mental model of the target tumor and its surrounding structures (e.g., organs, blood vessels, etc.). The surgeon’s task is to effectively recall the location of the tumor and mentally project it onto the patient in order to perform a successful surgery. If available, imaging modalities such as ultrasound, provide a live image to better locate the tumor and reduce the risk to the patient in the operating room. This makes the modeling and visualization a critical part of surgical planning.

Virtual Reality (VR) has received a lot of attention and is increasingly being used for surgical planning, surgery simulation, and in structural human anatomy^3–5^. The immersive experience provided by VR is claimed by televised medical series to allow surgeons to better navigate and discover problems inside organs or other internal structures. This blurs the actual reality. Indeed, although VR represents a key step toward enhancing the experience of learning and performing surgical techniques, there is an increased demand for objective assessment on whether VR is suitable for surgical planning. To our knowledge, there are no studies that have investigated the benefit of using VR for better spatial understanding of 3D models in surgical planning. This is especially true for evaluating the recall of medically relevant spatial information after the VR experience. VR technology provides an immersive experience and the ability to navigate and examine parts of an intricate and simulated three-dimensional scene. Our work is motivated by the fact that this technological advancement has not yet been evaluated using a task specific paradigm. One of the values of a virtual environment rendered in VR is the life-like simulation of surgical scenarios^6–9^. Anatomical data acquired during surgery can be integrated into such an environment by combining a range of imaging modalities and computational techniques^10^. This novel integration provides spatially and anatomically precise representations of medical image data^11^. However, it is important to quantify the actual contribution of VR to a task. Although non trivial, different studies addressed how such an environment should be used in this context. Within this scope, related works study the effects of frame rates, display latency, stereoscopic viewing, motion parallax, and the virtual reality type (i.e., head-mounted and stationary), respectively^12–15^. Findings from computer science studies have shown that VR displays combine visual cues such as motion parallax and stereopsis to help reveal structural information and improve learning tasks^13,15^. Moreover, in contrast to traditional displays, VR can combine visually immersive spatial data representations with our vestibular and proprioceptive senses^16^. Although the adoption of VR is ongoing across different fields (i.e., aeronautics, surgery, etc^17,18^), there is a growing need to validate whether there is a benefit from VR that can be transferred to the operating room.

In this paper, we examined whether VR could assist in accurate recall of intricate anatomical 3D structures by comparing memory of the location of a target structure between two learning conditions. The conditions are either afforded by a Head-Mounted Display (HMD), or by using a traditional Desktop screen or display (DT). The question was investigated by designing an experiment where participants navigated and learned about a three-dimensional scene showcasing a model and its internal structures. While a scene is often associated with multiple objects, we specifically refer to a scene as one object and its internal structures. For a more realistic approach, the study followed the sequence of events typically employed before (i.e., surgical planning) and during surgery in a tumor targeting paradigm: That is, creating the 3D model of the tumor, studying the model before surgery, recalling the position of the tumor during surgery, targeting it, and finally removing the tumor. Creating the model however was not part of the participants’ task as it is often prepared by medical experts and engineers before surgical planning. The two spatial tasks employed for evaluating the performance of participants were a scene understanding task and a direction estimation task. During the scene understanding task, participants were asked to explore a three-dimensional model and then assess the spatial relations of different structures in the model by filling a Multiple Choice Test (MCT) questionnaire. In the subsequent direction estimation task, participants were asked to point towards the position of a target structure (i.e., sphere) using a tracked pointing tool on a 3D printed version of the previously visualized model. To analyze the tasks, multiple quantitative metrics were used, recall accuracy, angular accuracy, confidence, and the usability. Our hypotheses for the different conditions were:

- Hypothesis 1: Participants will show higher recall accuracy and higher confidence ratings in the scene understanding task in the HMD condition as compared to the DT condition.
- Hypothesis 2: Participants’ targeting performance will be higher in the HMD condition in contrast to the DT condition.

Three main contributions of this study are: (1) We provide a work that fits into an evaluative reasoning and could prepare for future development of systematic evaluation frameworks^19^. (2) A controlled study comprising two interactive 3D visualizations across two learning conditions (HMD and DT) of two models (liver and pyramid). (3) We collected and analyzed participants’ preferences and their effectiveness in terms of recall accuracy and angular accuracy in the two models: Liver and Pyramid. All materials are available at https://www.github.com/ghattab/user-study. The experimental results of our study did not support either of our hypotheses.

## Related work

Related work addressed the educational effectiveness and viewing capabilities of VR systems in navigational and learning tasks. They have been conducted in two contexts, either in surgical planning and structural anatomy, or in other domains (e.g., spatial navigation, concept design).

### Surgical planning and structural anatomy

The use of VR has been found to enhance the surgeon’s spatial understanding of relevant anatomical structures. This is compatible with findings from many studies and different surgical procedures: heart surgery, aneurysm surgery, mandibular fracture related surgery, otologic or ear surgery^20–24^. Moreover, studies have shown that VR is of great educational value for learning different anatomy subjects in medical schools (e.g., heart, ear, brain, liver)^25–28^. A comprehensive study conducted by Huang et al.^29^ tested learners’ acceptance of VR for six different anatomy subjects. They found that immersion is the most important contributing factor to the perceived usefulness of VR. Even though modern technologies already exist, such as navigation during surgery (intraoperative), which enables the targeted location of non-visible structures, it must be said that these procedures are not used as standard and across the board. In most cases, preoperative visualization is limited to Computer Tomography (CT) or Magnetic Resonance Imaging (MRI), and both entities are usually displayed as two-dimensional slice images or 3D models. It is important to note that an integral part of surgical planning is the memorization of spatial information based on such data with the goal of building a mental model. This is a crucial step because it requires a high level of spatial understanding. Traditionally, surgeons rely on this ability when transferring the preoperative 3D model to the intraoperative scene or patient^5,30^. It is uncommon to view parallel images or a video feed while the surgeon is actively operating on the organ of interest. This leads to surgeons trying to memorize spatial relationships of internal structures. For example, anatomical features, relative positions, or tumor location. By relying on memory recall, then the surgeon relies on their mental model during surgery. Although there are many studies showing an advantage of providing a 3D model compared to visualizing only 2D tomography slices before surgery^31,32^, the comparison between an immersive HMD and a desktop display of the 3D model has not yet been performed.

### Other domains and areas of research

The related work spans almost all disciplines that employ computer-aided design such as engineering, architecture, physical modeling, and concept shape design^33–38^. User studies conducted by Mizell et al. concluded that VR helped users to successfully reconstruct an intricate 3D sculpture^39^. Such a task consists of comparing, perceiving spatial relationships, and matching the abstract shapes of the sculpture. In architecture, building design, and spatial navigation, Kuliga et al. found no significant difference in user performance when comparing user performance in a highly detailed virtual or a real environment^35^. Moreover, Tüzün and Ozdinc found that the decrease of distractors in a virtually rendered environment has a positive influence on the user’s conceptual and spatial learning^34^. For concept shape design, VR performs better in the context of requiring less time, fewer shape descriptors, and no specifications of exact dimensions^36,38^. Studies in spatial cognition that examined participants’ navigation skills and memory using either a HMD or DT have found that the HMD provided less unnecessary movements and a more accurate and confident recall of spatial information. Notably, a study concerned with memory palaces and the recall of large amounts of information found that the HMD allowed for superior recall of information^16^. In summary, it can be said that research efforts are aimed at achieving a better adaptation to human sensory abilities through the use of VR interfaces. Although VR technology is increasingly integrated into surgery, its benefit for relevant endpoints such as surgical tasks and outcome are currently unclear.

## Methods and materials

### Participants

Fifty-six volunteers (female = 29) with a mean age of 30.18 years took part in the experiment. Participants were students and medical staff recruited from the faculty of Carl Gustav Carus at Technische Universität Dresden and from the surrounding community. Prior to the start of the experiment, participants completed written informed consents about data sharing in accordance with the ethical standards. All participants had normal or corrected to normal vision and were screened for the presence of stereopsis using the Random Dot 2 LEA Symbols Stereoacuity Test by VAO. The study has a 2 (learning condition: HMD vs. DT) $\times$ 2 (model: Liver vs. Pyramid) between subjects design. The complete procedure a recruited participant follows is reported below in paragraph Procedure, while the Condition $\times$ Model is visually clarified in a diagram. Participants were randomly assigned to one of the four experimental groups: 16 participants were randomly assigned to the DT Liver combination, 13 participants were assigned to the HMD Liver combination, 15 participants were assigned to the DT Pyramid combination, and the remaining 12 participants were assigned to the HMD Pyramid combination. To investigate the possibility that medical participants might have an experiential advantage with respect to liver anatomy, the use of two very different structures, namely the anatomic model of a liver and the non-medical model of a pyramid, was chosen to minimize such bias. In addition, the comparison with non-medical participants is useful in that it allows us to examine the possible influence of the specific experience of medical professionals with respect to surgical planning or anatomy on spatial understanding.

### Materials

**Figure 1:**
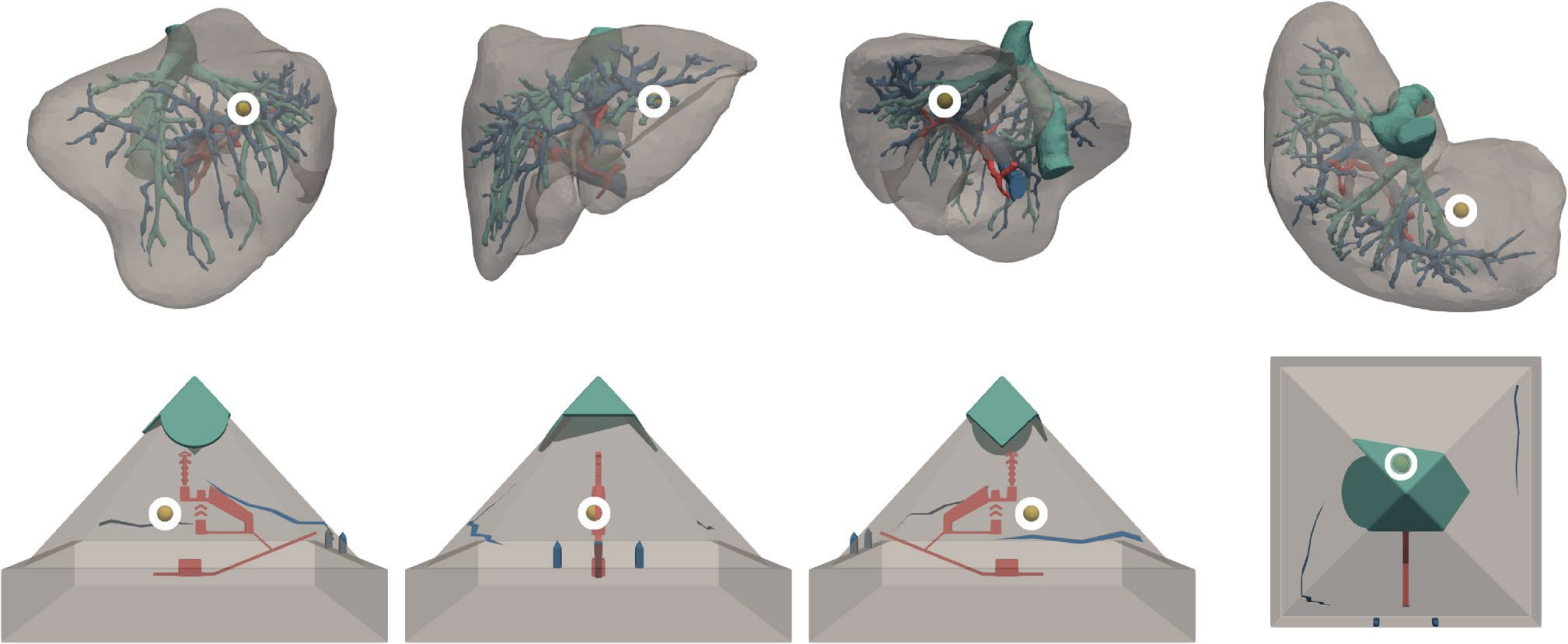
3D Visualizations. Each row represents a model and each column its visualization along an axis. The latter is displayed according to the following axes, from left to right: +x, +y, $-$x, $-$z. (Top) Liver: portal vein in blue (#4e79a7), hepatic artery in red (#e15759), hepatic vein or venous system in teal (#76b7b2). (Bottom) Pyramid: pyramid tip or pyramidion in teal, internal chambers in red, surface features (i.e., two cracks and entrance) in blue. The common encodings are: surface in light gray (#bab0ac), sphere in yellow (#edc948). To better perceive internal structures, surface opacity is set to 0.5 and the sphere is highlighted by a white circle.

In the experiment, 2 different models were used: a liver and a pyramid, respectively. Both models were used in virtual and real versions, respectively. The liver visualization was taken from the ircadb anatomical and medical image database depicting patient model number 17. The pyramid visualization represented a modified version of the model 296260 from Thingiverse. In order to provide further visual cues to the pyramid surface, we added additional features to the final visualization. That is to say, an entrance to the pyramid’s chambers, cracks on the four different faces of the pyramidion, and two obelisks. The visual properties of the visualizations such as color, size, texture, and transparency remained consistent across models, while respecting domain specific standards (e.g., hepatic artery in red)^40^. They are described in detail in Fig. 1. For both the liver and the pyramid, the main surface was depicted in gray whereas the sphere was yellow. Regarding the size, both models were scaled to a 1:1 ratio in the visualization. The texture varied depending on the visualization. For example, the liver’s texture remained unchanged to reproduce the real-world model of the selected ircadb liver. The pyramid’s texture however, was simplified in order to obtain a smooth and uniform surface. Both visualizations were assigned two transparency modes (a) opaque and (b) transparent (empirically set to 0.7) that could be switched by pressing a large opacity button. The rendering of the models and their viewing was possible thanks to an adapted version of the IMHOTEP framework^3,41^.

For the real world models, each visualization was materialized using the rapid prototyping technique in 3D printing technology. We used Poly Lactic Acid plastic (PLA) for the pyramid, and Acrylonitrile Butadiene Styrene (ABS) for the liver. Each model was scaled according to the liver patient size. Prior to the 3D printing, each model was mounted on a laboratory clamp stand holder for tracking purposes. After printing, the features of each visualization were painted using gouache paint (opaque and water soluble) for a faithful reproduction of each vis in Fig. 1 and were displayed at equal height from the base of the stand.

### Apparatus

In the learning condition HMD, participants viewed the 3D visualizations via an HTC Vive HMD with an 110$^{\circ }$ field of view and a refresh rate of 90 Hz. Interaction with the virtual scene was made via a controller in which trigger based events were mapped to navigation and button selection. A selection was possible by using a pointer or a shooting beam. In the learning condition DT, participants viewed the stimuli via a computer screen with a spatial resolution of $1920\times 1080$ pixels and a refresh rate of 60 Hz (ProLite B2480HS-B2 - iiyama). The computer was equipped with an NVIDIA GeForce GTX 1080 graphics cards and Intel i7-6700K 4.0 GHz processor. Interaction with the virtual scene was achieved in this condition with the use of a mouse, in which holding the button and moving the mouse permitted rotation and scaling of the scene. The interaction with each printed model was tracked between the participant and the printed model. This was achieved with the use of an infrared Polaris Spectra/Vega camera, passive trackers attached to each model of interest and the pointer tool. The pointer tool enabled the recording of the pointer position and its orientation in the local coordinate system.
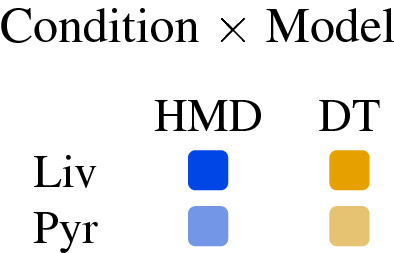


### Study procedure

**Figure 2:**
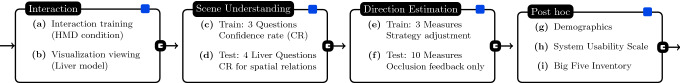
Example procedure for HMD-Liv group (). A participant P is assigned to view the Liver model in the HMD condition. (**a**) P learns how to interact with a demo model. (**b**) Then when viewing the assigned model, P is told that the sphere is the target. During Task 1: *Scene Understanding Task*, (**c**) A Multiple Choice Test (MCT) is used for confidence training. Correct answers to training questions are given and P is asked to reconsider strategy for Confidence Rating (CR). (**d**) Testing follows without feedback. During Task 2: *Direction Estimation Task* A Polaris tracking system is used to track P and the model of interest. (**e**) A tracked pointing tool is used to point at a point of entry (PoE) on the Liver printed model. P approaches the printed model as it is seen in the initial view in the visualization of the model in (**b**). Upon occlusion, P is asked to review the strategy (training only) (**f**) In testing, P restarts from the initial view to record all PoE with occlusion feedback only. (**g**) The demographics part included (Gender, age, etc). (**h**) Two System Usability Scale (SUS): one for the interaction in the HMD condition in which P interacted with the Liver model visualization, and another for the pointing/tracking system in which P interacted with the printed Liver model. (**i**) The Big Five Inventory (BFI-K) before P ended the study. To conform the procedure for each P, an internal checklist was used. It can be found at https://github.com/ghattab/user-study/blob/master/materials/study-related/study_checklist_en.pdf.

Each participant was screened for stereo vision using the Random Dot 2 LEA Symbols Stereoacuity Test by VAO, and afterwards was randomly assigned to either the HMD or the DT condition while viewing the pyramid or the liver respectively, forming 4 different combinations. An example workflow of the procedure for a participant in the HMD-Liv group () is shown in Fig. 2.

The experiment started with the Interaction Training in which participants were presented with a demo visualization in order to familiarize themselves with the controller or the mouse depending on the condition. Although participants were given ample time, the demo lasted for a maximum of five minutes and during this time participants were allowed to pan, rotate, zoom, and change the transparency of the demo visualization. The demo sequentially prompts the participant for each interaction and moves onto the next one—only and only if—the current one has been successfully completed. Therefore, when participants successfully completed this phase, the scene understanding task began. Participants were presented the model of interest (i.e., liver or pyramid) and were explicitly told about the existence of a target, i.e., sphere and that it is of importance to notice it. Depending on the type of the model, the target was introduced to participants either as a tumor in the case of the liver or as a treasure in the case of the pyramid model, respectively. Their task was to explore and study the position of the target as well as all features of the visualisation by using the interaction functions (e.g., rotate, zoom, transparency, etc) previously learned in the demo during Interacting training. There was no time limit for this exploration phase.

Afterwards, without seeing the model anymore, participants completed a questionnaire which was divided in two training questions and four testing questions. The training questions involved participants to identify the color of the sphere in both models, the number of vessel trees in the liver model, and the number of cracks in the pyramid’s surface. While answering each question, participants were then asked to provide a confidence level for that question which ranged from a scale of 1 to 10, with 10 being certain and 1 a result of random guessing. Participants were given the correct answer to both training questions. In the four testing questions for the scene understanding task, participants were asked to determine the spatial relationship of the objects presented in the 3D visualization using the following positioning keywords: left–right, above-below, in front and behind. Since the spatial dimensions of left–right, above-below can change after the interaction (e.g., rotation, transparency) with the models has occurred, participants were reminded to answer the questions while having in mind the default/initial viewpoint of the model as a reference point. For example, in the liver model participants had to indicate the position of a sphere in the model, as a potential tumor, relatively to the portal vein. A possible answer to this question could be: right, above, behind, using all three directions of every possible spatial dimension. In the pyramid model participants were asked to locate the sphere in the model, as a potential treasure. Its position was also central in the model, yet slightly to the back and on the left. It is worth mentioning that participants were neither informed about the sphere size, nor about the distances between the different objects within the scene. For the Pyramid, this coincided with the entrance. The test questions are reported below for the Pyramid.

- Q1. Where is the sphere from the upper chamber?
- Q2. Where is the sphere starting from the middle chamber?
- Q3. Where is the sphere starting from the lower chamber?
- Q4. Where is the sphere from the entrance?

The test questions are reported below for the Liver.

- Q1. Where is the sphere in the liver?
- Q2. Where is the sphere starting from the nearest branch of the hepatic veins (turquoise blue)?
- Q3. Where is the sphere starting from the artery (red)?
- Q4. Where is the sphere from the rift on the right side of the liver?

After completing the scene understanding task, participants proceeded to the direction estimation task. Here, the task was to interact with the printed models, i.e., to target the location of an internal object, that is estimating the location of a sphere on the printed models of the pyramid and liver using a tracked pointing tool. Before starting training, participants were informed that the real-world models are positioned in the same default or front view that they were initially experienced in. In the training phase, participants had to point at three predetermined points of entries in order to familiarize themselves with the tracked pointing tool. That is, they were instructed to correctly position the tracked pointing tool and direct it at the target object. Indeed, the goal of the training is to figure out how they should position themselves to target the sphere while allowing for the tracking system to see both the model and pointer positions. In some instances, this resulted in changing their approach and adapting for the system to track both the model and the pointer without any occlusion. In the testing phase, participants pointed to 10 points of entries sequentially while we were also recording their responses via the tracking system. For the pyramid model, entry points were randomly sampled on three accessible lateral facets (excluding the facet with tracking and mounting devices). For the liver model, we explicitly compared the difficulty of the entry points to the pyramid model, and selected them based on expert opinion (experienced surgeon in liver surgery). At the end of study, participants were required to fill a posthoc questionnaire. It comprised three forms: a demographic and experience form, a system usability scale or SUS form, and a Big Five Inventory form or BFI-K (K = 20 items)^29,42,43^. For the system usability scale (SUS) participants provided their responses to 10 questions (see Supplementary Material for SUS questions) with a given 1–5 scale to fill (e.g., 1 strongly disagree and 5 strongly agree with the statement).

### Ethics consideration

All protocols and procedures were approved by University Hospital Carl Gustav Carus Institutional Review Board (No. EK 230062018) and all methods were performed in accordance with these guidelines and regulations, which included all users providing informed consent prior to participation. All responses were collected using an in-house software, and all testing was completed during one session.

### Data curation

Rule-based data validation was performed through the entire data to ensure data integrity and data consistency^46^.

## Overview of statistical methods

### Performance in scene understanding task

Performance in the Scene Understanding task was assessed by taking into account (i) the accuracy in the recall of a target located within the model, and (ii) the confidence in this recall. The assessment was performed using four questions, each referring to a certain aspect of the position of the target in three spatial dimensions. For each correctly recalled dimension, one point was awarded to the question score. In contrast, for each incorrectly recalled dimension, one point was taken off the question score. Therefore, the by-question accuracy score ranged from $-$3 to 3 points.

The effects of learning condition and type of model on accuracy and confidence was analysed using linear mixed effects models. For both models with accuracy and confidence as the dependent measures, a random intercept was included, and learning condition (HMD vs. DT) and type of model (Liver vs. Pyramid) were entered as predictor variables, while controlling for the Question number (i.e., Q1–Q4).

### Performance in direction estimation task

Targeting performance in the direction estimation task was measured by using the angle (in degrees) between the tracked pointing tool and the ray going from the pointer origin towards the printed model (Liver or Pyramid). The effects of learning condition and type of printed model on participants’ angular accuracy were estimated using a multiple linear regression model with target distance, learning condition (HMD vs. DT), and type of printed model (Liver vs. Pyramid) as predictor variables.

### System usability evaluation

The overall HMD and DT system usability was assessed with the System Usability Scale (SUS)^44^. This was done to assess the subjective usability of each learning environment, which to our knowledge has not been evaluated in the past. The System Usability Scale is based on ten questions. The answers to these questions were given on a five-point Likert scale, which afterwards was converted to a single SUS score according to the formula: 2.5 * [(Sum of scores from Question 1, 3, 5, 7, 9 $-$ 5) + (25 $-$ Sum of scores from Questions 2, 4, 6, 8, 10)]. Given the question structure, the formula ensured that the total score would range from 0 to 100. The usability scored less than 51 was considered “awful” , from 51 to 68—“poor”, 68 to 80.3—“good” and above 80.3—“excellent”. SUS scores were calculated separately for the *Learning condition* and the *Direction Estimation* task.

## Results

### Scene understanding task

#### Effects of learning condition and model type on recall accuracy

Results from the linear mixed effects model showed that although the learning condition (HMD vs. DT) did not have a significant effect on the recall accuracy, $F(1,53) = 0.0058, p\,=\,0.95$, there was a significant effect for the model type (Liver vs. Pyramid), $F(1,53) = 15.2209, p < 0.001$, that differed across Questions 1–4, $F(3, 162) = 8.0906, p < 0.001$. We report the mean *M* and the Standard Error *SE*, respectively. Notably, participants who were assigned in the Pyramid group ($M= 1.03$, $SE= 0.15$) were more accurate than those assigned in the Liver group ($M= 0.19$, $SE= 0.14$), $t(53) = 3.901, p < 0.001$. Results also revealed a significant interaction between the model type (Liver vs. Pyramid) and Question (1 to 4), $F(3, 162) = 8.0906, p < 0.001$.

Post hoc comparisons with adjusted *p* values showed responses in Question 1 were more accurate for participants in the Pyramid group ($M=1.25$, $SE=0.23$) compared to the those in the Liver group ($M=0.03$, $SE= 0.22$). The difference was found to be significant, $t(180) = 3.758, p < 0.001$. This assumes that the first question was comparable for both models. In the Pyramid, Q1 was “Where is the sphere from the upper chamber?”, while in the Liver Q1 was “Where is the target in the liver”. This corresponded to a mental view from above in the Pyramid (upper chamber), while in the Liver model, this was the default or initial view of the visualization (front). Indeed, all test questions were designed in parallel for each model while bearing in mind a similar difficulty among the models.

Similarly in Question 4, participants assigned in the Pyramid model were significantly more accurate in their responses ($M= 0.95$, $SE= 0.23$) than the participants assigned in the Liver model ($M=-0.75$, $SE= 0.22$. The difference was found significant, $t(180) = 5.169, p < 0.001$.

Altogether, the results indicate that simple internal structures consisting of simple geometrical shapes like the ones included in the pyramid model were represented and retrieved from memory more easily compared to intricate structures such as the anatomical and internal parts of a patient liver model.

**Table 1:** Linear mixed-effects models for Accuracy and Confidence in Recall of the Scene Understanding Task.

ine	Accuracy			Confidence		
	Estimate	SE	*p*	Estimate	SE	*p*
ine Intercept	0.02	0.24	0.91	7.88	0.44	**0.00**
HMD	0.01	0.21	0.93	0.17	0.48	0.72
Pyramid	1.22	0.32	**0.00**	−0.55	0.56	0.32
Question 2	0.58	0.27	**0.03**	−1.68	0.34	**0.00**
Question 3	0.86	0.27	**0.00**	−3.06	0.34	**0.00**
Question 4	−0.79	0.27	**0.00**	−2.72	0.34	**0.00**
Pyramid: Question 2	−1.40	0.40	**0.00**	1.72	0.48	**0.00**
Pyramid: Question 3	−0.60	0.40	0.13	2.88	0.48	**0.00**
Pyramid: Question 4	0.45	0.40	0.25	1.87	0.48	**0.00**

Each dependent measure is modelled as a function of the predictors: Learning Condition (with DT as reference category), type of model (with Liver as the reference category), Question (with Question 1 as a reference category) and their interaction. For each fixed effects and interaction, we report the coefficient and its standard error, along with the associated *p*-value. Statistically significant predictors (at the $\alpha = .05$ level) are in bold.

#### Effects of learning condition and model type on confidence

Results from the mixed effects regression model showed that the levels of confidence in participants’ responses did not differ between the learning conditions (HMD vs. DT), $F(1,53) = 0.1259, p = 0.72$, as shown in Table 1. Similar to recall accuracy, results showed higher confidence levels for participants assigned in the Pyramid group ($M= 7.17$, $SE= 0.34$), than for participants assigned in the Liver group ($M= 6.10$, $SE = 0.33$) with $p< .05$. The difference was found significant, $t(53) = 2.207, p\,=\,0.03$. Moreover, results revealed the presence of a significant interaction between and the model type (Liver vs. Pyramid) and Question (1–4), $F(3,162) = 11.9488, p < .001$. Specifically, the confidence values were significantly higher for Questions 2, 3 and 4 for participants assigned in the Pyramid group than those assigned in the Liver group. The corresponding test statistics were $t(97.2) = 2.060, p\,=\,0.04$, $t(97.2) = 4.099, p < 0.001$, $t(97.2) = 2.317, p = 0.02$. For Question 1, the difference was not significant, $t(97.2) = 0.982, p = 0.33$. Taken together, the findings indicated that the model type had an impact on participants’ confidence in their responses. This suggests that, internal structures such as those found in the liver model, not only hinder the retrieval of structural information from memory, but also affect the confidence to what is being actually retrieved.

### Direction estimation task

The initial analysis of angular accuracy in the direction estimation task was performed using a linear mixed-effect model with random intercept and with the target distance, learning condition (HMD vs. DT) and the model type (Liver vs. Pyramid) as fixed effects. Between-subject variability, estimated using the R library nlme, was rather low, as the SD estimate was about 0.0014, which indicated that a multiple linear regression model with the same fixed effects may also be appropriate. In fact, the AIC of the latter model was found to be 4883.7, as compared to AIC of 4884.487 of the mixed-effects model. Likelihood ratio test between these two models led to the same conclusion, $\chi ^2(1) = 9.50582e-07$, p = 0.9992. Therefore, for the final analysis, the multiple linear regression model was used. Analysis of variance entailed that the target distance and model were significant predictors, with $F(1,555) = 47.4051, p < 0.001$, and $F(1,555) = 68.2107, p < 0.001$, respectively. The effect of learning condition and the interaction between this condition and model were not statistically significant, $F(1,555) = 1.0711, p = 0.30$, and $F(1,555) = 0.0841, p = 0.77$, respectively. These results are reported in Table 2. In particular, participants assigned in the Pyramid group were found to be less precise in their targeting of the sphere ($M= 59.8$, $SE= 1.21$), than participants assigned in the Liver group ($M= 45.5$, $SE =1.16$). The difference was found significant, $t(555) = 8.188, p < .001$.

**Table 2:** Multiple linear regression model for Angular Accuracy of the Direction Estimation Task.

ine	Angular accuracy		
	Estimate	SE	*p*
ine Intercept	38.63	2.16	**0.00**
Target distance	0.01	0.00	**0.00**
HMD	2.36	2.22	0.29
Pyramid	14.78	2.25	**0.00**
HMD * Pyramid	−0.92	3.20	0.77

Each dependent measure is modelled as a function of the predictors: Target Distance, Learning Condition (with DT as reference category), the model type (with Liver as the reference category) and their interaction. For each fixed effects and interaction, we report the coefficient and its standard error, along with the associated *p*-value. Statistically significant predictors (at the $\alpha = .05$ level) are in bold.

It is worth mentioning that on average, participants in the DT learning condition were more precise in their targeting ($M =51.7$, $SE=1.07$), than those in the HMD learning condition ($M=53.6$, $SE=1.19$). Although this was not significant, $t(555) = -1.181, p = 0.24$.

The difference between the two learning conditions was larger for participants in the Liver group (DT participants were on average 2.36 degrees more precise than HMD, $t(555) = -1.060, p = 0.29$), and smaller for participants in the Pyramid group (DT participants were, on average, 1.43 degrees more precise, $t(555) = -0.619, p = 0.54$). Finally, the variability captured by the overall linear model was not high numerically, $R^2$= 0.17, therefore the model’s explanatory value was low. Further results are reported in Tables S6, S7, and S8 for Precision Angle, Accuracy, and Confidence, respectively.

### System usability scale scores

System usability scores were calculated separately for the scene understanding and direction estimation tasks. Results are presented in the tables below.

For the scene understanding task, about 61% of participants found the system usability of the rendering of the visualizations to be excellent. While for the direction estimation task, about 59% of participants found the system usability of the tracking system to be good or excellent. Further details are reported in the Supplementary Material. In particular, the descriptive summaries of study endpoints with respect to the medical experience of participants are reported in Tables S3–S8.

## Discussion

The purpose of the present study was to evaluate whether VR technology is suitable for spatial understanding in surgical planning and medical learning by comparing performance across two learning conditions. Specifically, in one experiment, we examined whether participants would accurately represent and recall the internal structure of two rendered models, a pyramid and a liver, experienced either via a HMD or a DT. The results revealed that the learning condition (HMD or DT) did not influence participants’ memory and confidence ratings of the models shown in VR. In contrast, the model type , that is, whether the model to be recalled was a liver or a pyramid, significantly affected participants’ memory about the internal structure of the model. Furthermore, we investigated participants’ ability to accurately locate an internal object—a target sphere—by placing and directing a pointer tool on the printed models. The data indicated that localizing the internal position of the sphere was also unaffected by whether participants had previously experienced this particular model via HMD or DT. These results provide novel insights on the use of VR in a surgical planning scenario and also shed light on the mental model people create to recall internal spatial structures.

Contrary to our initial hypothesis, the participants were neither more accurate, nor confident when they experienced the models via HMD compared to when experienced via DT display. This is supported by the finding that participants reported similar confidence and exhibited similar performance across the learning conditions. Moreover, the learning condition where the initial encoding of information about the structure of the models took place, did not affect targeting performance later on in the direction estimation task. This result is in contrast with previous research on navigation showing that HMD compared to DT displays, enhanced the recall of spatial information due to the elevated sense of immersion that Head-Mounted Displays can offer^16^. Immersion in that research might have helped in structuring and remembering spatial information on the basis of reference cues such as landmarks and one’s egocentric viewpoint. However, results from our study demonstrate that immersion might not be an important factor in remembering internal anatomical structures.

The fact that immersion did not facilitate performance is reflected in participants’ lower recall accuracy and confidence in the liver model compared to the pyramid model. It may be that models, such as the liver, that do not have salient features are difficult to represent in memory, unlike objects with clearly defined geometric structures. Although specific internal structures exist, for instance the hepatic vein, it is possible that lower accuracy and confidence may be due to the difficulty that is introduced by the liver and its internal structures (e.g., distances across blood vessels that are naturally full of twists and turns). This result, questions findings from past literature that highlight the benefits of VR in surgery and medical learning^29^. According to past studies, immersive VR can aid surgeons to understand intricate anatomical structures for surgical planning^21,29^ due to its interactive interface and by providing a better visualization experience through the examination of an organ from multiple angles. However, the absence of an interaction between the learning condition and the model type challenge these previous findings and suggest that inspecting and interacting with the scene through multiple viewing angles while being immersed in a virtual environment does not provide any additional benefit over viewing the scene on a screen through the traditional DT. Although VR has progressively been used in various surgical applications, our findings show that this technology has no significant impact on medical learning and recall of acquired spatial knowledge in the context of surgical planning. Therefore, medical institutions should be cautious in using this technology with the prospect of improving learning outcomes and achieving better surgical planning.

Based on the results from the scene understanding task, one might expect participants to also perform better in the direction estimation task with the pyramid model. Surprisingly, we observed the reverse pattern of results; i.e., the pyramid yielded more error in angular accuracy than the liver did. First, a possible explanation is that the result was caused by medical experts being overall more experienced with printed models of liver during their medical education^2^. However, additional analyses taking into account participants’ medical expertise (see Supplementary Material) showed that this possibility is unlikely. Participants with more experience had a tendency to be more precise but this difference was not significant for the liver model. Perhaps the fact that the sample of experts (27) was smaller than the sample of non-experts has masked any profound differences in performance. Second, another explanation for this result is that participants with prior experience in VR may have an advantage as they benefited from both the pyramid’s simplicity and their experience. However, the results from extra analyses (see Supplementary Material) showed this explanation is also unlikely since VR expertise did not play an important role in performance in the direction estimation task. Third and a more interesting possibility, is that this result is due to differences in the encoding strategies that participants used based on the structural properties of each model. According to the Categorical Adjustment Model^45^, people carry out two levels of spatial encoding; a fine-grain level that contains precise metric information about objects’ locations, and a coarse level which relies on categorical information about objects’ locations. Since our models differ in form and in structure, it might be that for complex forms—with no obvious symmetry—such as the liver form and its internal structures, participants used a more fine-grained metric estimate to encode the target’s location. Indeed, we hypothesized that different geometries might lead to different types of information encoding. In this case, we believe that the features of the liver model are more pronounced: the ridge, the asymmetrical shape, etc. In contrast, the pyramid has a symmetrical shape. In contrast, for symmetrical objects like the pyramid, which can be divided into geometrically distinct segments, participants might have relied on categorical estimates (i.e., upper left, bottom right), to memorize the target’s location. Provided the pyramid features (i.e., obelisk, entry, cracks, and the pyramidion) were not well noted, a misidentification between front and back could easily lead to lower accuracy. Although both metric and categorical information can be combined to achieve maximal accuracy of judgment, often the use of the coarse level encoding introduces a bias towards a central value that occurs from using categories to correct the memory of non-precise metric measures^45^. This latter point may explain the less accurate performance with the pyramid compared to the liver model in the direction estimation task, where pointing with precision was required. In other words, it could be that relying on coarse categorical estimates rather than fine-grained estimates during encoding results in poor performance in the directional estimation task.

Although our study included a fair number of medical experts, future studies could examine whether a larger sample of people with medical expertise would alter the pattern of the observed results. It could be that people who are familiar with anatomical structures produce fewer errors in recall and targeting performance compared to non-experts. Moreover, further research may shed light on whether encoding of spatial information about complex objects shows better performance when such forms and their internal structures may be compared.

Overall, the findings from the present study provide new insight on the use of VR in surgical planning and medical learning, that to our knowledge has not been investigated before. We particularly defined two tasks, i.e., scene understanding and direction estimation, to record and compute various evaluation metrics. In particular, results showed similar recall performance and confidence across both learning conditions, suggesting that the use of VR does not necessarily lead to better understanding and/or learning of complex structures. This finding has important implications for surgical planning and medical education, where surgeons and students study anatomical structures of organs and are required to recall spatial information to create accurate mental representations. Although previous studies favored the use of immersive VR for surgical understanding of anatomical structures, the current results challenge previous findings and should be considered prior to learning tasks in medicine. We believe this is important because our results indicate that VR technology is still a major challenge for implementation in medical scenarios. Moreover, findings from the current study contribute a clear understanding on how complex anatomical structures might be represented in memory. Future experiments could compare performance on memory recall and angular accuracy of two structures that vary in their difficulty.

## Supplementary information

Supplementary Information 1.

## Data Availability

All data supporting the findings of this study and the employed models are available within the article and its Supplementary Information files. All data, models, photographs of the setup, figures, and in-house software are available at http://www.github.com/ghattab/user-study.
