# Supplementary material for: Investigating the utility of VR for spatial understanding in surgical planning: evaluation of head-mounted to desktop display
Source: Sci Rep. 2021 Jun 29;11:13440. doi: 10.1038/s41598-021-92536-x (PMC8241863; doi:10.1038/s41598-021-92536-x)
Supplement: Supplementary file 1 — Supplementary Information 1. [file 41598_2021_92536_MOESM1_ESM.pdf]

Supplementary Material

Investigating the utility of VR for spatial understanding in surgical planning: Evaluation of head-mounted to desktop display

Hattab and Hatzipanayioti *et al.*

In follow up analyses, we also explored the effects of medical experience and VR experience on performance in the scene understanding and direction estimation tasks. The goal of these exploratory analyses was to examine whether there is an advantage in performance for people who are more familiar with structures such as the liver compared to people who are naive to it. Similarly, we aimed to identify whether people with more VR experience would exhibit better performance in the HMD learning condition than people who are less experienced with this type of system. To do so, we explored separate models for two subsets of data with participants categorized based on their medical and VR experience, on recall accuracy, confidence, angular accuracy, and system usability scores.

Scene Understanding Task

Based on the linear regression model with the overall accuracy from Questions 1-4 as response and medical experience and model as predictors, neither medical experience nor its interaction with the model were significant predictors, $F(1,52) = 0.8860, p = 0.35$ and $F(1,52) = 0.4243, p = 0.518$, respectively. On average, participants with no prior medical experience were less accurate than those with some experience, $t(52) = -1.072, p = 0.29$. Under an analogous model with VR experience, neither VR experience nor its interaction with the model were not significant, $F(1,52) = 0.5468, p = 0.46$, $F(1,52) = 0.8591, p = 0.36$. 0n average, participants without VR experience were less accurate, $t(52) = -0.315, p = 0.75$.

In the similar modeling setup, but with the average confidence from Questions 1-4 as response, both medical experience and its interaction with the model were found marginally significant: $F(1,52) = 3.7007, p = 0.06$, $F(1,52) = 5.4146, p = 0.02$, respectively. Participants with no prior medical experience were overall less confident, $t(52) = -1.910, p = 0.06$, in recall of their corresponding training objects. Among participants without medical experience, those in the liver group were significantly less confident than those in the pyramid group, $t(52) = -3.378, p = 0.001$, while among experienced participants the difference was not significant, $t(52) = 0.158, p = 0.88$. Neither VR experience nor its interaction with the model were found significant predictors of

the recall confidence, $F(1, 52) = 0.0523, p = 0.82$, $F(1, 52) = 0.0019, p = 0.97$, respectively.

As seen in Table S1, VR experience was not a significant predictor for the direction estimation task.

Effect	Estimate	S.E.	p.value
ine Intercept	77.391	4.468	0
VR experience, 2 to 4 years	0.516	8.827	0.954
VR experience, < 2 years	4.229	4.912	0.393
HMD	−19.132	6.213	0.003
Pyramid	−5.353	5.946	0.372
HMD * Pyramid	16.244	8.766	0.07

Table S1: Regression Model for Direction Estimation Task. Reference levels are DT, Liver, none of VR experience.

System Usability Scale Scores

Under the regression model for the scene understanding task, the VR experience was found to be a marginally significant predictor of SUS ($F = 3.2962$, $p = 0.05$). For example, the participants without any VR experience gave the lowest average SUS (M = 78.7, SE = 1.75), while the average SUS for the most experienced participants was the highest (M = 90.9, SE = 5.34), $t(50) = -2.179, p = 0.08$. The interaction between learning condition and training model was not found significant, $F(1, 50) = 1.2261$, $p = 0.27$. On average, participants in the pyramid group found the system more useful, $t(50) = 2.154, p = 0.04$.

On the other hand, the estimated average difference in SUS between the learning condition and the model type, was almost negligible, $t(50) = 0.224, p = 0.82$, and therefore, the data did not provide any evidence that HMD training was found to be more useful for scene understanding.

In the analogous regression analysis of SUS in the direction estimation task, both VR experience and the model were not significant, $F(1, 50) = 0.0366, p = 0.96$ and $F(1, 50) = 0.1848, p = 0.67$. The interaction between the learning condition and the model was marginally significant ($F(1, 50) = 3.434$, $p = 0.07$). Under this regression model, the learning condition was found the only significant predictor of SUS ($F(1, 50) = 6.296$, $p = 0.02$), the DT group found their learning condition more useful, $t(50) = 2.520, p = 0.02$. For example, the average usability score of the participants in the DT-Liver subgroup was on average higher than in the HMD-Liver subgroup, $t(50) = 3.079, p = 0.003$. As in the scene understanding task, the Pyramid group, gave on average, higher usability scores, $t(50) = 0.617, p = 0.54$. However, because the regression model itself explained only about 17 % of variability in SUS, the conclusions are rather insubstantial (see Table S2).

Effect	Estimate	S.E.	p.value
ine Intercept	77.427	2.846	0
VR experience, 2 to 4 years	12.251	5.622	0.034
VR experience, < 2 years	8.137	3.129	0.012
HMD	-3.713	3.957	0.353
Pyramid	3.07	3.787	0.421
HMD * Pyramid	6.182	5.583	0.273

Table S2: Regression Model for System Usability Scale for the scene understanding task. Reference levels are DT, Liver, no VR experience.

	DT		HMD	
Med Experience (years)	Liver	Pyramid	Liver	Pyramid
ine 0	7	11	9	5
less than 2	3	0	1	1
2 to 4	3	0	0	2
4 to 6	0	1	1	1
6 to 10	1	3	1	0
over 10	2	0	1	3

Table S3: The number of participants with the respective medical experience in each of the four study subgroup.

Med Experience ine	Method	Model	n	Mean	95% CI
0	DT	Liv	7	83.60	71.30 - 95.90
0	DT	Pyr	11	81.40	74.50 - 88.30
0	HMD	Liv	9	78.10	68.90 - 87.20
0	HMD	Pyr	5	80.00	62.60 - 97.40
less than 2	DT	Liv	1	70.00	
less than 2	DT	Pyr	3	90.00	77.60 - 102.00
less than 2	HMD	Liv	1	75.00	
2 to 4	DT	Liv	3	87.50	75.10 - 99.90
2 to 4	HMD	Liv	1	82.50	
2 to 4	HMD	Pyr	1	100.00	
4 to 6	DT	Liv	3	73.30	37.50 - 109.00
4 to 6	HMD	Pyr	2	92.50	60.70 - 124.00
6 to 10	DT	Pyr	1	75.00	
6 to 10	HMD	Liv	1	82.50	
6 to 10	HMD	Pyr	1	92.50	
Over 10	DT	Liv	2	81.20	1.84 - 161.00
Over 10	HMD	Liv	1	85.00	
Over 10	HMD	Pyr	3	80.80	60.90 - 101.00

Table S4: SUS for the learning condition by medical experience. CI: Confidence Interval. n: sample size.

Med Experience ine	Method	Model	n	Mean	95% CI
0	DT	Liv	7	74.30	60.00 - 88.60
0	DT	Pyr	11	76.60	69.70 - 83.40
0	HMD	Liv	9	66.40	54.70 - 78.10
0	HMD	Pyr	5	78.50	62.60 - 94.40
less than 2	DT	Liv	1	75.00	
less than 2	DT	Pyr	3	70.00	13.10 - 127.00
less than 2	HMD	Liv	1	20.00	
2 to 4	DT	Liv	3	81.70	39.40 - 124.00
2 to 4	HMD	Liv	1	45.00	
2 to 4	HMD	Pyr	1	55.00	
4 to 6	DT	Liv	3	84.20	74.70 - 93.70
4 to 6	HMD	Pyr	2	55.00	-263.00 - 373.00
6 to 10	DT	Pyr	1	45.00	
6 to 10	HMD	Liv	1	62.50	
6 to 10	HMD	Pyr	1	87.50	
Over 10	DT	Liv	2	80.00	-15.30 - 175.00
Over 10	HMD	Liv	1	62.50	
Over 10	HMD	Pyr	3	65.80	56.30 - 75.30

Table S5: SUS of the tracking system by medical experience. CI: Confidence Interval. n: sample size.

Med Experience	Method	Model	n	Mean	95% CI
ine					
0	DT	Liv	70	42.80	38.50 - 47.10
0	DT	Pyr	110	61.40	57.70 - 65.20
0	HMD	Liv	90	44.70	41.40 - 47.90
0	HMD	Pyr	50	64.70	59.80 - 69.60
less than 2	DT	Liv	10	46.50	31.00 - 62.10
less than 2	DT	Pyr	30	59.00	51.40 - 66.60
less than 2	HMD	Liv	10	49.80	32.00 - 67.60
2 to 4	DT	Liv	30	45.50	38.50 - 52.50
2 to 4	HMD	Liv	10	40.40	29.40 - 51.30
2 to 4	HMD	Pyr	10	64.20	51.90 - 76.50
4 to 6	DT	Liv	30	41.50	34.50 - 48.40
4 to 6	HMD	Pyr	20	55.70	45.10 - 66.20
6 to 10	DT	Pyr	10	48.10	29.80 - 66.40
6 to 10	HMD	Liv	10	62.20	47.80 - 76.50
6 to 10	HMD	Pyr	10	59.80	44.40 - 75.20
Over 10	DT	Liv	20	42.30	33.80 - 50.80
Over 10	HMD	Liv	10	40.60	29.70 - 51.60
Over 10	HMD	Pyr	30	60.60	52.40 - 68.70

Table S6: Angular accuracy by medical experience. CI: Confidence Interval. n: sample size.

Med Experience ine	Method	Model	n	Mean	95% CI
0	DT	Liv	28	-0.25	-0.69 - 0.19
0	DT	Pyr	44	1.11	0.81 - 1.42
0	HMD	Liv	36	0.25	-0.26 - 0.76
0	HMD	Pyr	20	0.75	0.14 - 1.35
less than 2	DT	Liv	4	1.25	-1.14 - 3.64
less than 2	DT	Pyr	12	1.75	0.98 - 2.52
less than 2	HMD	Liv	4	1.00	-2.67 - 4.67
2 to 4	DT	Liv	12	0.08	-0.66 - 0.82
2 to 4	HMD	Liv	4	0.25	-2.14 - 2.64
2 to 4	HMD	Pyr	4	1.50	0.58 - 2.42
4 to 6	DT	Liv	12	0.25	-0.69 - 1.19
4 to 6	HMD	Pyr	8	0.62	-0.98 - 2.23
6 to 10	DT	Pyr	4	-0.50	-2.09 - 1.09
6 to 10	HMD	Liv	4	0.50	-1.09 - 2.09
6 to 10	HMD	Pyr	4	1.50	-0.09 - 3.09
Over 10	DT	Liv	8	0.50	-0.39 - 1.39
Over 10	HMD	Liv	4	0.25	-1.27 - 1.77
Over 10	HMD	Pyr	12	1.00	0.01 - 2.01

Table S7: Accuracy in recall by medical experience. CI: Confidence Interval. n: sample size.

Med Experience ine	Method	Model	n	Mean	95% CI
0	DT	Liv	28	5.32	4.27 - 6.37
0	DT	Pyr	44	6.84	6.19 - 7.50
0	HMD	Liv	36	5.17	4.40 - 5.93
0	HMD	Pyr	20	8.10	7.33 - 8.87
less than 2	DT	Liv	4	6.50	4.45 - 8.55
less than 2	DT	Pyr	12	7.42	6.39 - 8.45
less than 2	HMD	Liv	4	6.50	5.58 - 7.42
2 to 4	DT	Liv	12	7.25	6.23 - 8.27
2 to 4	HMD	Liv	4	7.00	4.75 - 9.25
2 to 4	HMD	Pyr	4	10.00	10.00 - 10.00
4 to 6	DT	Liv	12	6.75	6.14 - 7.36
4 to 6	HMD	Pyr	8	4.25	2.26 - 6.24
6 to 10	DT	Pyr	4	5.50	4.58 - 6.42
6 to 10	HMD	Liv	4	7.25	2.50 - 12.00
6 to 10	HMD	Pyr	4	6.00	3.75 - 8.25
Over 10	DT	Liv	8	6.88	5.36 - 8.39
Over 10	HMD	Liv	4	10.00	10.00 - 10.00
Over 10	HMD	Pyr	12	8.42	7.32 - 9.52

Table S8: Confidence in recall by medical experience. CI: Confidence Interval. n: sample size.
